# The Beneficial Effects of Lactobacillus Strains on Gut Microbiome in Alzheimer’s Disease: A Systematic Review

**DOI:** 10.3390/healthcare13010074

**Published:** 2025-01-03

**Authors:** Michael Quansah, Monique Antoinette David, Ralph Martins, Emad El-Omar, Silvana Mirella Aliberti, Mario Capunzo, Slade O. Jensen, Mourad Tayebi

**Affiliations:** 1 Neuroimmunology Laboratory, School of Medicine, Western Sydney University, Campbelltown, NSW 2560, Australia; m.quansah@westernsydney.edu.au (M.Q.); m.david@westernsydney.edu.au (M.A.D.); 2 Department of Medicine and Therapeutics, Medical School, University of Ghana, Accra LG25, Ghana; 3 Macquarie Medical School, Faculty of Medicine, Health and Human Sciences, Macquarie University, Macquarie Park, NSW 2109, Australia; ralph.martins@mq.edu.au; 4 Microbiome Research Centre, School of Clinical Medicine, UNSW Medicine & Health, St George & Sutherland Clinical Campuses, UNSW, Kogarah, NSW 2217, Australia; e.el-omar@unsw.edu.au; 5 Hygiene and Preventive Medicine Section, Department of Medicine, Surgery and Dentistry “Scuola Medica Salernitana”, University of Salerno, Baronissi, 84081 Salerno, Italy; sialiberti@unisa.it (S.M.A.); mcapunzo@unisa.it (M.C.); 6 School of Medicine, Microbiology and Infectious Diseases, Ingham Institute for Applied Medical Research, Western Sydney University, Liverpool, NSW 1871, Australia; s.jensen@westernsydney.edu.au

**Keywords:** probiotics, Alzheimer’s disease, mild cognitive impairment, gut microbiota

## Abstract

Background/Objectives: Growing evidence suggests that the gut–brain axis influences brain function, particularly the role of intestinal microbiota in modulating cognitive processes. Probiotics may alter brain function and behavior by modulating gut microbiota, with implications for neurodegenerative diseases like Alzheimer’s disease (AD). The purpose of this review is to systematically review the current literature exploring the effects of probiotic supplementation on gut microbiota and cognitive function in AD and mild cognitive impairment (MCI). Methods: A comprehensive literature search was conducted across PubMed/Medline, Embase, and Scopus to identify relevant randomized controlled trials (RCTs) from inception to 20 August 2024. The search focused on comparing outcomes between intervention and control/placebo groups. Data searches, article selection, data extraction, and risk of bias assessment were performed in accordance with Cochrane guidelines. Systematic Review Registration: PROSPERO registration no: CRD42023446796. Results: Data from four RCTs involving 293 Individuals (AD and MCI patients) receiving mainly *Lactobacillus and Bifidobacterium* strains showed some beneficial effects on cognitive function, altered gut microbiota composition, and positively affected metabolic biomarkers. However, variability in microbiota assessment across studies limits the interpretation of results. The limited number and quality of the existing studies make it difficult to draw definitive conclusions from the data. Additional high-quality research is clearly needed. Conclusions: Probiotics show promise as an adjunctive intervention for cognitive decline, but larger, long-term trials are needed to confirm their efficacy and clinical applicability in neurodegenerative diseases like AD.

## 1. Introduction

Alzheimer’s disease (AD) is the most common form of dementia worldwide, accounting for 60% to 70% of all cases [1,2]. With improvements in living conditions and a rising global life expectancy, the prevalence of AD is expected to triple by 2050, resulting in substantial social and economic costs [2,3]. Recognizing the urgency of this issue, the World Health Organization (WHO) has classified dementia as a major public health priority. In response, the World Health Assembly developed the Global Action Plan on the Public Health Response to Dementia 2017–2025, which aims to enhance the quality of life for individuals with dementia and their caregivers while alleviating the burden on societies and economies. One of the primary objectives is to reduce the risk of dementia by promoting healthy dietary patterns [2]. The plan also emphasizes the need for evidence-based interventions that are accessible to most populations, encouraging individuals to adopt healthier lifestyles and reduce exposure to modifiable risk factors, with the aim of curbing the disease’s rapid increase [4]. Moreover, it was posited that Alzheimer’s might be caused as a consequence of the interaction between genetic, macro-environmental, and micro-environmental factors [5,6,7,8,9].

The precise cause of AD remains unknown, but several theories suggest factors contributing to its development. One of the most widely accepted explanations is the amyloidogenic hypothesis, which postulates that the accumulation of extracellular amyloid-beta (Aβ) deposits plays a key role. However, numerous studies aiming to support this hypothesis have failed to achieve successful clinical outcomes, raising significant doubts about its validity [10,11]. Genetic research has identified four genes involved in the development of AD: amyloid precursor protein (APP), presenilin 1 (PS1), presenilin 2 (PS2), and apolipoprotein E (ApoE) [11]. The metabolism of APP by β- and γ-secretases results in the formation of Aβ fragments, which accumulate and form toxic amyloid plaques (AP) [11]. In addition to AP, AD is characterized by the deposition of intracellular neurofibrillary tangles (NFTs), composed mainly of hyperphosphorylated Tau protein. Normally, Tau protein stabilizes microtubules and facilitates the transport of substances along axons, however. Abnormal aggregation of Tau leads to neuronal death in AD [12].

Given the gradual progression of AD, early intervention strategies are critical [13]. Over the past decade, several randomized controlled trials (RCTs) have demonstrated promising outcomes using dietary interventions, particularly probiotics—live beneficial microorganisms—and prebiotics—non-digestible fibers that promote the growth of these microorganisms–to slow the progression of Alzheimer’s disease (AD) [14]. Regulation of the gut–brain axis has garnered significant attention as a promising therapeutic approach for neurodegenerative disorders, including Alzheimer’s disease (AD) [15]. This axis, which facilitates bidirectional communication between the central nervous system and the gastrointestinal tract, is influenced by complex interactions involving gut microbiota, neural pathways, immune responses, and metabolic signaling [16]. Dysregulation of the gut–brain axis has been implicated in the progression of AD, with evidence suggesting that gut microbiota alterations contribute to neuroinflammation, amyloid-beta deposition, and synaptic dysfunction [17] and recent research has focused on devising more targeted interventions that account for alterations in gut microbiota in AD patients, opening new possibilities for managing disease progression [18].

### 1.1. Gut Microbiome

The human microbiome consists of a diverse group of microorganisms that live in symbiosis with the host, playing essential roles in regulating health and disease. These microorganisms can have neutral, beneficial, or harmful effects on the host. Remarkably, almost 99% of the genes present in the human body are microbial, amounting to more than 10 million genes [19,20]. Distinct types of bacteria inhabit specific regions of the human body, predominantly residing on/in the skin, eyes, respiratory system, urogenital system, and gastrointestinal tract.

The gut microbiome (GM) is the most abundant microbial community associated with the body, comprising about 95% of the total human microbiome [20]. Most microorganisms reside in the distal part of the gastrointestinal tract, as the stomach’s hydrochloric acid along with bile and pancreatic secretions, inhibit bacterial colonization in the upper gut. This creates a bacterial concentration range of 10^1^ to 10^3^ CFU/mL. As one moves from the jejunum to the ileum, the bacterial population in the small intestine gradually increases to 10^4^ to 10^7^ CFU/mL. In the colon, the concentration reaches an estimated 10^11^ to 10^12^ CFU/mL per gram of intestine [21]. Advances in next-generation sequencing technologies over the past decade, along with developments in bioinformatics have enabled more precise and cost-effective analyses of the microbiome’s composition. This progress has significantly enhanced our understanding of the microorganisms that inhabit the human gastrointestinal tract [22]. The two dominant phyla in the gut are Bacteroidetes and Firmicutes, which together constitute 70–75% of the GM. A balanced distribution of genera within these phyla is crucial for a healthy microbiota. For example, within the Firmicutes phylum, the presence of *Lactobacillus* spp. is considered more beneficial for health compared to those of *Clostridium* or *Enterococcus* [22]. Other phyla, such as Proteobacteria and Actinobacteria are present in smaller proportions, while fungi, viruses, archaea, and protozoa make up about 1% of the microbiome [23].

While the host’s genetic DNA remains relatively stable throughout life, the composition of the microbiome is highly dynamic and constantly changing [24]. Several factors, particularly early-life events like the mode of birth (vaginal or caesarean) can influence the microbial composition [25,26]. In addition, factors such as age, development, diet, medication use (especially antibiotics), smoking, lifestyle, genetics, and disease can significantly shape the GM [27].

Studies have shown a strong correlation between the GM and cognitive and mental impairment. For example, germ-free mice and antibiotic-induced dysbiosis models have exhibited altered production of metabolites critical for cognitive functions, such as brain-derived neurotrophic factor (BDNF), gamma-aminobutyric acid (GABA), N-methyl-D-aspartate (NMDA) receptors, and tryptophan [28]. Research on disorders characterized by cognitive impairment including Parkinson’s Disease (PD), AD, Schizophrenia, and Major Depressive Disorder (MDD) has revealed that alterations in GM composition may contribute to the development and progression of these conditions [29]. Therefore, targeting the GM might present a promising avenue for influencing cognitive health in both clinical and non-clinical studies [30].

### 1.2. Gut–Brain-Axis

In the 1880s, William James and Carl Lange were the first to propose the concept of two-way communication between the central nervous system (CNS) and the gut suggesting that this connection plays a role in regulating emotions. This idea was later expanded by physiologist Walter Bradford Cannon, who emphasized the brain’s significant role in gastrointestinal function [31]. Although the link between the gut and brain has been recognized for a long time, it was not until the last few decades that researchers began to adopt a broader perspective of the human body. This shift started in the 1990s when the effectiveness of treating gastric issues solely with drugs, without considering the brain’s involvement, was called into question [32,33]. This more comprehensive approach to medicine signifies a growing understanding of the microbiome’s impact on clinical treatments [34].

The gut–brain–microbiome axis involves a complex network of communication between the brain, GM, and immune responses. This axis operates through multiple bi-directional pathways, including the vagus nerve, enteroendocrine cells, and metabolites that influence neurotransmitter production [35]. These pathways regulate a range of processes, from inflammation and appetite to more complex behaviors, such as social isolation or repetitive movements [36]. The GM appears to influence the brain by altering axis signaling through bile acids [37], inflammatory markers [37,38], and metabolites [38,39], which in turn affect critical neurochemical pathways. For instance, certain bile acids have been linked to brain volume, Aβ deposition, and the eventual development of AD [40].

### 1.3. Probiotics

Given the influence of the GM on brain function, modifying its composition through probiotic supplementation may offer protection against cognitive decline. Probiotics are live microorganisms that, when administered in adequate amounts, provide health benefits to the host [36]. Fermented foods such as sauerkraut, pickles, yogurt, and miso naturally contain beneficial microbes that can act as probiotics [41].

One bacterial genera that has demonstrated a positive impact on cognitive disorders is *Lactobacillus*. A key component of the human GM, *Lactobacillus* has gained attention for its potential to slow the progression of AD, due to its anti-inflammatory [42]. One of the critical mechanisms is the regulation of cytokine and chemokine secretion, which helps maintain immune balance and suppress chronic inflammation. *Lactobacillus* can induce anti-inflammatory cytokines, such as interleukin-10 (IL-10), while downregulating pro-inflammatory cytokines, including tumor necrosis factor-alpha (TNF-α) and interleukin-6 (IL-6).

This modulation often occurs through the nuclear factor kappa B (NF-κB) signaling pathway, a key regulator of inflammation. Lactobacillus interacts with toll-like receptors (TLRs) on host epithelial and immune cells, leading to either suppression or controlled activation of the NF-κB pathway.

*Lactobacillus* is a Gram-positive bacterium that produces lactic acid, widely recognized for its beneficial effects on gut health. Studies show that *Lactobacillus* spp. can increase bacterial diversity creating competition with harmful bacteria for space and nutrients. This limits the overgrowth of pathogens and helps maintain a balanced GM [42]. Consequently, it prevents gastrointestinal inflammation, which is an early indicator of chronic diseases, including AD [43].

Additionally, *Lactobacillus* spp. strengthen the intestinal epithelial barrier by improving intercellular connections, thereby preventing harmful substances from entering the bloodstream [44]. By fermenting dietary fibers, *Lactobacillus* spp. produce beneficial metabolites like short-chain fatty acids (SCFAs), including butyrate, acetate, and propionate [45]. Notably, butyrate has been shown to improve cognitive function and reduce AD-like symptoms in animal models [46].

Among the various species, *Lactobacillus rhamnosus GG* has demonstrated protective effects on intestinal epithelial cells and can lower inflammatory markers like interleukin-8 (IL-8) [47]. It has also been linked to improvements in metabolic factors, including glucose tolerance, insulin sensitivity, and inflammation [48,49,50]. These findings suggest that *L. rhamnosus GG* may positively influence brain health and cognitive function. Moreover, *L. rhamnosus* GG supplementation has been linked to reductions in anxiety-like, obsessive-compulsive disorder-like, and depressive behaviors in mice, alongside a decreased risk of neuropsychiatric disorders in children.

Preclinical research further supports the potential of specific *Lactobacillus* spp. in enhancing cognitive function. In one study, the introduction of *Lactobacillus plantarum* improved memory and learning in mice with Alzheimer’s, likely by modulating SCFA-related pathways [51]. Another study found that *Lactobacillus gasseri* reduced neuroinflammation in AD-affected mice by decreasing microglial activation and pro-inflammatory cytokine production, highlighting its anti-inflammatory effects [52].

Oral administration of probiotics can directly modify the GM by increasing the abundance and diversity of beneficial microbes. This can lead to changes in metabolite production, reduced inflammation, and improved gut barrier function, while also influencing the hypothalamic-pituitary-adrenal (HPA) axis [53]. Through these mechanisms, probiotics offer a promising way to modulate the central nervous system (CNS) and are being explored as treatments for various CNS-related disorders [54]. Probiotics are also being investigated for their potential to enhance cognitive function in non-clinical populations. Studies in mice have shown that both single-strain and multi-strain probiotics improve spatial and non-spatial memory [54].

This review aims to systematically examine randomized clinical trials in human subjects to determine whether probiotics can mitigate or improve cognitive decline associated with dementia or pre-existing cognitive impairment.

## 2. Materials and Methods

### 2.1. Search Strategies and Selection Criteria

This systematic review was conducted in accordance with the PRISMA 2020 guidelines [55], with the corresponding PRISMA checklist displayed in Table 1. The review was registered in the International Prospective Register of Systematic Reviews (PROSPERO) under registration number CRD42023446796. To identify relevant studies, comprehensive searches were conducted in Pubmed/Medline, Embase, and Scopus, covering publications up to 20 August 2024. The search strategy, detailed in Table 1, employed a combination of keywords and Boolean operators. It was constructed using a combination of natural language and structured terms (MeSH), following the PICO framework (Table 2).

### 2.2. Eligibility Criteria

Randomized controlled trials (RCTs) were included based on the PICO framework outlined in Table 2. Eligible studies met the following criteria:

- RCTs conducted in patients diagnosed with Alzheimer’s disease (AD) or mild cognitive impairment (MCI).
- Intervention involving probiotics, prebiotics, or synbiotics.
- Comparison of the intervention against a control or placebo group.
- Main outcomes focused on cognitive function (measured by validated scale) and gut microbiota diversity and composition.

Additional outcomes included changes in metabolic variables, inflammatory biomarkers, and oxidative stress markers.

The titles and abstracts of identified studies were imported into EndNote Web for duplication assessment. After removing duplicates, the remaining studies were screened based on the inclusion and exclusion criteria using Covidence. Full-text versions of eligible studies were retrieved, and reasons for exclusion were documented. Two reviewers (Michael Quansah and Mourad Tayebi) independently assessed the titles and abstracts. Any discrepancies were resolved through discussions between the two reviewers.

### 2.3. Data Extraction

Two independent reviewers (M.Q. and M.T.) extracted data from the eligible studies, cross-checking for discrepancies. For qualitative analysis, the following data were collected:

- Reference;
- Country of study;
- Participant details;
- Probiotic strain used;
- Duration of intervention;
- Dose and dosage regimen;
- Data collection tools;
- Primary and secondary outcomes;
- Main results.

The primary outcomes focused on changes in general cognitive function (memory, attention, language, orientation, and learning) in AD and MCI patients. The MMSE and MoCA are cognitive screening tools commonly used in clinical and research settings to assess global cognitive function. The MMSE focuses on orientation, attention, memory, language, and visuospatial skills, while the MoCA offers a broader assessment, particularly in detecting mild cognitive impairment. These tools were used in the included studies as they provide standardized and comparable measures for cognitive outcomes. Secondary outcomes included biochemical parameters such as synaptic plasticity, oxidative stress markers, and enzyme levels.

### 2.4. Risk of Bias and Quality Assessment

The risk of bias was assessed according to the Cochrane Handbook for Systematic Reviews of Interventions [50], focusing on the following domains:

- Random sequence generation;
- Allocation concealment;
- Blinding of participants and personnel;
- Blinding of outcome assessment;
- Incomplete outcome data;
- Selective outcome reporting;
- Other potential sources of bias.

Each domain was rated as high, low, or unclear risk. The risk of bias assessment was performed independently by two reviewers (M.Q. and M.T.), and any disagreements were resolved through discussion.

## 3. Results

### 3.1. Search Results and Selection of Studies

**Figure 1:**
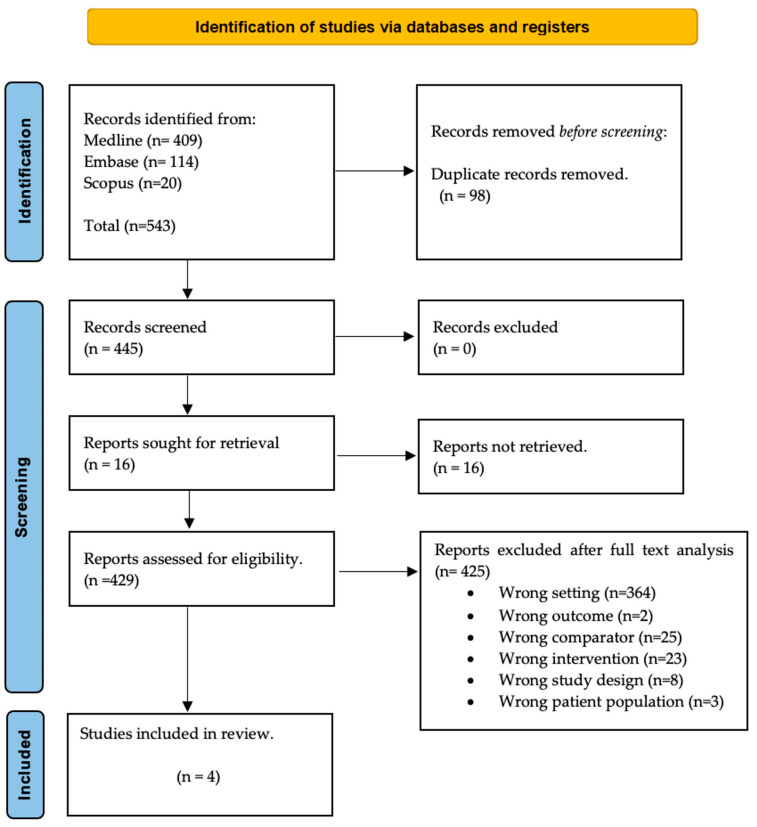
Flow diagram illustrating the identification of studies for inclusion.

The initial search across three databases identified 543 articles. After removing duplicates, 445 articles remained for screening (Figure 1). Following the screening of titles and abstracts, 429 articles were further assessed for full-text eligibility. Of these, 425 articles were excluded for not meeting the inclusion criteria, as detailed in Figure 1. Consequently, four randomized controlled trials (RCTs) were included in the qualitative analysis.

### 3.2. Characteristics of Clinical Studies

The four randomized controlled trials (RCTs) included in this review were conducted between 2018 and 2023, focusing on individuals diagnosed with Alzheimer’s disease (AD) or mild cognitive impairment (MCI). Collectively, these studies enrolled 293 participants (138 with AD and 155 with MCI), aged between <60 and 90 years. A slight predominance of male participants was observed across the trials.

The geographical distribution of the studies is as follows: two were conducted in Iran [56,57], one in Japan [58], and one in China [59]. The probiotic interventions utilized various formulations from the Lactobacillus and Bifidobacterium genera, recognized for their potential neurocognitive benefits through modulation of the gut–brain axis. Three studies employed multi-strain formulations [56,57,59], while one utilized a single-strain probiotic [58]. The duration of these interventions ranged from 12 weeks [56,57,59] to 24 weeks [58], with daily probiotic dosages varying from 3 × 10^9^ CFU to 10^15^ CFU.

Probiotic delivery methods included encapsulated probiotics in three studies [56,57,58], while one study used powdered formulations [59]. Cognitive assessments were conducted using validated tools, notably the Mini-Mental State Examination (MMSE) and the Montreal Cognitive Assessment (MoCA). These tools were selected for their ability to assess cognitive domains relevant to AD and MCI populations. Additionally, one study [58] incorporated the Japanese version of the Alzheimer’s Disease Assessment Scale–Cognitive Subscale (ADAS-Jcog), which provides a more nuanced evaluation of cognitive changes.

Secondary outcomes included assessments of body mass index (BMI), oxidative stress markers, metabolic profiles, and inflammatory cytokines, providing insights into potential mechanisms underlying probiotic efficacy. Furthermore, two studies [56,59] evaluated changes in gut microbiota composition, offering evidence of microbiota modulation as a potential mediator of cognitive benefits. Table 3 and Table 4 summarize the detailed characteristics of the included studies, including study design, participant demographics, diagnostic criteria, interventions, and outcomes.

### 3.3. Qualitative Synthesis

Primary Outcomes: Cognitive Function

The cognitive outcomes associated with probiotic interventions were mixed, reflecting variability in participant characteristics, probiotic formulations, and intervention durations:

- Akhgarjand et al. [57] reported significant improvements in MMSE scores, reduced anxiety (GAD-7), and enhanced instrumental activities of daily living (IADL) among participants with mild to moderate AD. However, basic activities of daily living (ADL) were unaffected.
- Fei et al. [59] documented significant improvements in MMSE scores, recall, and calculation abilities among participants with MCI. Additionally, sleep quality, as measured by the Pittsburgh Sleep Quality Index (PSQI), improved significantly in the probiotic group.
- Asaoka et al. [58] observed enhancements in specific cognitive subdomains such as attention and executive function using the ADAS-Jcog. However, total cognitive scores did not show significant changes, suggesting that probiotic benefits may be domain-specific.
- In contrast, Agahi et al. [56] found no significant cognitive improvements in participants with advanced AD, 85% of whom had severe disease. The authors suggested that the severity of neurodegeneration in this population limited the potential for therapeutic effects, highlighting the importance of disease stage in evaluating the efficacy of probiotic interventions.

Secondary Outcomes: Biomarkers and Gut Microbiota

The findings for secondary outcomes varied across studies, reflecting heterogeneity in study populations and probiotic formulations:

- Akhgarjand et al. [57] and Fei et al. [59] reported improvements in metabolic and inflammatory markers, including reductions in BMI and inflammatory cytokines. Fei et al. also observed significant enhancements in sleep quality.
- Asaoka et al. [58] reported suppression of brain atrophy, as evidenced by MRI scans, but found inconsistent effects on BMI and oxidative stress markers.
- Agahi et al. [56] observed no significant changes in metabolic or inflammatory markers, likely due to the advanced disease stage in their participants.

Regarding gut microbiota composition, only two studies [56,59] evaluated this parameter:

- Fei et al. [59] reported increased microbiota diversity, particularly in *Lactobacillus* and *Bifidobacterium* abundance, which was associated with cognitive improvements.
- Asaoka et al. [58], however, found no significant alterations in gut microbiota composition, suggesting that probiotic effects on the microbiome may depend on strain specificity and baseline microbiota characteristics.

Heterogeneity in Outcomes

The variability in outcomes reflects the influence of several factors, including probiotic strain selection, dosage, intervention duration, and participant characteristics:

- Studies using multi-strain formulations (e.g., Fei et al. [59]) reported more consistent improvements in cognitive and metabolic outcomes compared to single-strain interventions (e.g., Asaoka et al. [58]).
- Differences in disease severity were also critical; for instance, advanced AD stages (Agahi et al. [56]) appeared less responsive to probiotics than earlier stages (Akhgarjand et al. [57], Fei et al. [59]).

Explanation of MMSE and MoCA Usage

The MMSE and MoCA assessments were chosen for their widespread validation and ability to evaluate multiple cognitive domains, including memory, attention, language, and executive function. These tools are particularly suited for detecting changes in cognitive function associated with probiotic interventions:

- The MMSE is commonly used to assess global cognitive function and track longitudinal changes in AD populations.
- The MoCA is more sensitive to mild cognitive impairment, making it a preferred tool for MCI populations.

### 3.4. Risk of Bias Assessment

**Figure 2:**
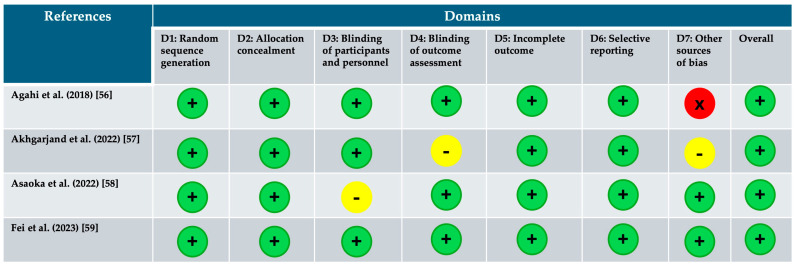
Risk of bias assessment for the included studies. Risk of bias summary: Green (+): Low risk of bias; Yellow (-): Unclear risk of bias; and Red (X): High risk of bias. [56,57,58,59].

**Figure 3:**
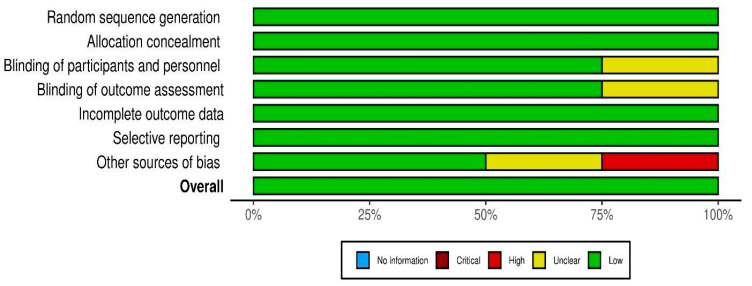
Risk of bias assessment for the included studies. Risk of bias graph.

The risk-of-bias assessment results for each included study are illustrated in Figure 2 and Figure 3. All four studies were double-blinded randomized clinical controlled trials. They were classified as having a low risk of selection and conduct bias. However, Akgarjand et al. presented unclear blinding of participants and personnel [57], while Asaoka et al. lacked clarity in describing the blinding of outcome assessors, which could introduce selection and detection biases, respectively [58]. Agahi et al. reported a high risk of other sources of bias [56]. In summary, Figure 2 and Figure 3 evaluates the risk of bias based on seven domains (D1 to D7), with the following judgments: (i) Green (+): Low risk of bias; (ii) Yellow (-): Unclear risk of bias; and (iii) Red (X): High risk of bias. Domains Assessed:

**D1: Random sequence generation**—Evaluates if the randomization process was adequately performed.

**D2: Allocation concealment**—Assesses if the allocation to intervention/control groups was concealed.

**D3: Blinding of participants and personnel**—Checks if participants and personnel were blinded to intervention groups.

**D4: Blinding of outcome assessment**—Evaluates if outcome assessors were blinded.

**D5: Incomplete outcome data**—Assesses the handling of missing data.

**D6: Selective reporting**—Checks if all pre-specified outcomes were reported.

**D7: Other sources of bias**—Considers other potential sources of bias.

**Study-Specific Observations**:

**Agahi et al. (2018) [56]:**

○ All domains, except D7, have a low risk of bias.
○ **D7 (Other sources of bias):** High risk of bias, making the overall judgment high.

**Akhgarjand et al. (2022) [57]:**

○ Low risk of bias for most domains.
○ **D3 and D4:** Unclear risk of bias, but overall judgment is low.

**Asaoka et al. (2022) [58]:**

○ Low risk of bias for most domains.
○ **D3:** Unclear risk of bias, but overall judgment is low.

**Fei et al. (2023) [59]:**

○ All domains show a low risk of bias.
○ Overall judgment: Low risk of bias.

**Interpretation:**

- **Low Risk Studies:** Fei et al. (2023) [59], Akhgarjand et al. (2022) [57], and Asaoka et al. (2022) [58] have predominantly low-risk judgments, making them reliable sources of evidence.
- **High-Risk Study:** Agahi et al. (2018) [56] has a high risk of bias due to other sources of bias (D7). Caution is required when interpreting its findings.
- **Areas Needing Clarification:** For Akhgarjand et al. (2022) [57] and Asaoka et al. (2022) [58], domains related to blinding (D3 and D4) need better reporting or methodology to ensure reliability.

This figures suggests that, while most studies are of high quality, further methodological details for D3 and D4 in some studies and addressing the concerns in D7 for Agahi et al. (2018) [56] could improve confidence in the findings.

## 4. Discussion

This systematic review aimed to determine whether intake of probiotics can minimize the risk of MCI and AD. Overall, the evidence presented supports the use of probiotics to enhance cognition, with all included studies reporting improvements in at least one cognitive measure. Enhanced cognition was consistently observed in individuals with MCI across two clinical groups. Notably, substantial improvements in Mini-Mental State Examination (MMSE) scores were replicated in a randomized controlled trial involving subjects with lower baseline scores [56]. Two clinical trials that utilized the MMSE to evaluate cognitive function in individuals with AD and MCI also indicated significant cognitive improvement following probiotic intervention compared to placebo [57,59].

In contrast, a clinical trial by Agahi et al. utilizing the Test Your Memory (TYM) test showed no significant effect [56], possibly due to various variables such as differences in study populations, strain efficacy, and intervention duration. The lack of improvement in cognitive function observed in Agahi et al.’s study could be attributed to several factors. First, the severity of cognitive impairment in their participants may have been more advanced, which might have limited the potential for cognitive improvements. Second, the strain-specific efficacy of the probiotics used in their trial might not have been optimal for improving cognitive function. Third, the study had a short intervention duration of just 12 weeks, which might not have been long enough to observe meaningful cognitive changes, particularly in individuals with more advanced stages of MCI or AD. Additionally, factors such as comorbid conditions, medication use, or other lifestyle variables may have influenced the outcome.

The three studies employing a 12-week intervention with multi-strain probiotic supplements demonstrated improvements in cognitive function overall, with the exception of Agahi’s study [56,57,59]. While the preliminary evidence appears promising, further studies are needed to draw definitive conclusions. Future clinical trials should be conducted for longer than 12 weeks to track the progression of AD and MCI and ascertain whether probiotics are more effective in the early stages or when cognitive impairment is more severe.

One study found improvements in recall, attention, calculation, visual-spatial skills, and executive function, providing robust evidence that memory and learning abilities were enhanced by probiotic intervention in older MCI patients over 12 weeks. Additionally, the studies suggested that probiotic intervention improved sleep quality and gastrointestinal symptoms [59]. Although some individuals reported improvements in Montreal Cognitive Assessment (MoCA) scores, it may not be suitable for healthy adults, as it serves primarily as a quick screening tool for MCI and dementia, like MMSE. Asaoka et al. conducted a 24-week intervention that demonstrated probiotic intake suppressed the progression of brain atrophy, as indicated by VSRAD based on brain MRI scans, and resulted in increased gut microbiome diversity [58].

Of the four studies, three utilized one or two strains or species, demonstrating improvements in cognitive function [56,58,59]. However, one study that employed a single strain as a probiotic supplement showed some improvement in specific subscale scores, but not in the overall score following probiotic intake in MCI. Fei et al. used a multi-strain probiotic formulation with different genera and species, achieving significant increases in MMSE scores, attention, recall, and calculation scores, along with notable improvements in MoCA scores, decreased Pittsburgh Sleep Quality Index (PSQI) scores, and enhanced gastrointestinal symptom rating scale (GSRS) total scores in the probiotic group. However, no significant differences were noted at the phylogenetic level between the probiotic and placebo groups [59].

The studies included both *Lactobacillus* and *Bifidobacterium* as single-strain supplements and as part of diverse combinations in multi-strain supplements. Among these two genera, *Lactobacillus* spp. have shown clinical efficacy in impacting cognitive function. For instance, *Lactobacillus* spp. (e.g., *L. plantarum*) exhibit superior survival and colonization rates in the human gastrointestinal tract compared to *Bifidobacterium* spp. [60]. Previous research has indicated that Lactobacillus species possess anti-inflammatory properties, including reducing intestinal barrier permeability, increasing short-chain fatty acid levels, and restoring brain-derived neurotrophic factor levels in cognitively impaired individuals [61]. Lactobacillus spp. have been shown to modulate inflammation through various mechanisms. Specifically, these probiotics can reduce levels of pro-inflammatory cytokines such as TNF-α and IL-6 while increasing levels of anti-inflammatory cytokines like IL-10. These effects are mediated through the NF-κB signaling pathway, a key regulator of inflammation. Additionally, Lactobacillus spp. promote the production of short-chain fatty acids (SCFAs), which contribute to maintaining intestinal barrier integrity and further reduce intestinal permeability.

Limited information regarding the specific strains used in multi-strain supplements complicates the determination of which combinations are most effective for enhancing cognitive performance. Although some studies suggest that conflicts between strains may impair the efficacy of multi-strain probiotic supplements, no direct evidence links this to cognitive outcomes [62]. Even when strains inhibit each other in a combined environment, efficacy may not necessarily decrease, and in some instances, combinations may outperform individual strains due to synergistic or additive effects [63]. To maximize the efficacy of probiotic interventions for cognitive health, understanding the mechanisms of action and interactions between strains is crucial.

Prominent psychobiotics, including *Bifidobacterium* and *Lactobacillus* spp., have been extensively studied [64,65]. However, not all single probiotics exhibit psychobiotic potential, highlighting the need for improved screening methods to create effective probiotic strategies for AD, given that the positive impacts of probiotics can be strain-specific.

The International Scientific Association for Probiotics and Prebiotics (ISAPP) defines probiotics as providing health benefits when consumed in adequate doses. However, it does not specify an effective dose or frequency for probiotic therapy. Various organizations, including ISAPP [65], Health Canada [66], the World Gastroenterology Organisation [67], and the Italian Ministry of Health (IMH) [68], have made efforts over the past decade to establish recommended probiotic dosages.

To ensure safety and efficacy, the IMH recommends a minimum of 1 × 10^9^ CFU per day for food-based probiotics and dietary supplements. Although all four studies included in this review utilized a daily dose of 1 × 10^9^ CFU or more, it remains unclear whether this dose is optimal for improving cognitive function. Some studies have tested higher doses (up to 10^10^ CFU/day) and observed greater cognitive improvements. However, the ideal dosage may vary depending on factors such as the specific probiotic strain, population characteristics, and study duration. Future research should explore whether higher doses or different dosing schedules lead to more significant cognitive benefits.

The duration of intervention in existing literature ranges from 12 weeks to 24 weeks. While a 3-week probiotic intervention has shown some health benefits [68], it may be too short to determine its impact on cognition. Based on the limited evidence available, no definitive conclusions can be drawn from this review.

Our systematic review has several limitations. First, despite comprehensive literature searches, we may have overlooked qualified studies. Second, specific characteristics of the included research may pose potential risks of bias due to commercial funding and design flaws. These factors restrict the generalizability and strength of the conclusions that can be drawn from this review. Moreover, while some studies have examined the impact of modifications to gut microbiota on cognitive performance, few have conducted fecal analyses to assess microbiota composition post-intervention, and none have collected pre-intervention samples. Evaluating the fecal microbiome before and after the intervention could provide insights into how the intervention modifies the composition of resident gut microbes. Although these data are valuable, current research indicates that probiotic therapies are unlikely to yield significant alterations in microbiota composition, particularly among healthy individuals, in terms of both diversity and richness [69]. It may be more insightful to investigate how probiotics stabilize and reinforce the microbiota rather than substantially altering its composition [70]. In addition, clinical studies could explore the effects of other microbiota modulators, such as prebiotics, synbiotics, or fecal transplantation, given that these strategies are almost unexplored.

## 5. Conclusions

This systematic review suggests that taking probiotics for at least 12 weeks may enhance cognitive performance in individuals with MCI or AD, likely through modulation of the gut microbiome. However, the results of the included studies remain inconclusive, highlighting the need for high-quality, long-term studies to confirm their efficacy in AD and MCI. Moreover, additional research is required to better understand the neuroprotective potential of probiotics at various stages of AD, as current RCTs lack definitive evidence. To determine the effectiveness of probiotic interventions in improving cognitive function in specific populations, well-designed and controlled RCTs focusing on both cognitive performance and underlying mechanisms are essential. Such research could provide valuable insights for clinical applications and the personalized use of probiotics to support cognitive health.

## Figures and Tables

**Table 1:** Search strategies used in each database.

Database	Search Strategy
PubMed Medline	(Probio OR Bifidobacter OR Lactobac OR lactic acid bacteria) (Title/Abstract) OR Probiotics/OR exp Bifidobacterium/OR exp Lactobacillus/AND (microbiome OR microbiota OR intestinal flora OR gut dysbiosis) (Title/Abstract) OR Alzheimer Disease/
Embase	Lactobac (Title/Abstract) OR exp Lactobacillus/AND (microbiome OR microbiota OR intestinal flora OR gut dysbiosis) (Title/Abstract) OR Alzheimer Disease/
Scopus	(lactobac) (Title/Abstract) AND (microbiome OR microbiota OR intestinal AND flora OR gut AND dysbiosis) (Title/Abstract) AND (alzheimer) (Title/Abstract)

Note: Keywords, Boolean operators, and search syntax applied in Medline, Embase, and Scopus.

**Table 2:** PICOS criteria for inclusion and exclusion of studies.

Parameters	Inclusion	Exclusion
Participants	Adults diagnosed with AD or MCI using any established diagnostic criteria.	Adults with AD or MCI without a probiotic intervention or placebo.
Intervention	Probiotics, synbiotics, or prebiotics administered orally or enterally, with no restriction on strains, doses, or duration.	Studies involving other therapeutic interventions or drugs combined with probiotics.
Comparison	Comparison with placebo or no intervention.	N/A
Outcomes	Improvement in cognitive function (using a validated cognitive test), amyloid-beta PET scans, gut microbiome diversity, metabolic and inflammatory biomarkers.	N/A
Study Characteristics	RCTs published after 2014.	Animal studies, cell lines, case reports, reviews, editorials, comments, and letters.

**Table 3:** Key Characteristics of Included Clinical Studies.

Study Type	Population	Diagnostic Criteria	P	C	Age (P)	Age (C)	Gender (P)	Gender (C)	Intervention	Duration	Primary Outcome	Secondary Outcome	Main Findings
Double-blind RCT [56]	48 AD individuals, aged 65–90	NINDS-ADRDA	25	23	79.70 (1.72)	80.57 (1.79)	7/18	10/13	Probiotic, 2 capsules/day	12 weeks	TYM	TAC, GSH, MDA, IL-6, IL-10, TNF-_, 8-OHdG, NO, BMI	85% of patients had severe AD. No significant changes in cognitive function or metabolic indicators after probiotic intake
Double-blind RCT [57]	90 mild/moderate AD, aged 50–90	MMSE, CFT	60	30	67.93 (7.8)	67.77 (7.9)	32/28	16/14	Probiotics, 2 capsules/day	12 weeks	MMSE	BMI, ADL, IADL, GAD-7	Probiotic improved cognitive status, anxiety, and IADL but had no effect on ADL
Double-blind RCT [58]	115 MCI, aged 65–89	MMSE, CDR	55	60	77.2 (5.8)	78.9 (4.3)	26/29	25/35	Probiotic, 1 capsule/day	24 weeks	ADAS-Jcog	BMI, MMSE, MRI	Probiotics improved some cognitive subscales but not total scores. VSRAD scores showed suppressed brain atrophy
Double-blind RCT [59]	40 MCI, aged < 60	MMSE, MoCA	20	20	76.40 (9.61)	75.30 (9.75)	10/11	11/10	Probiotic, 2g/day	12 weeks	MMSE, MoCA	Diabetes, BMI, Hypertension	Probiotic group had higher MMSE, recall, and calculation scores, and improved sleep quality (PSQI). No difference in gut microbiota abundance

Notes: RCT: Randomized controlled trial; CFU: Colony-forming units; AD: Alzheimer’s disease; MCI: Mild cognitive impairment; MMSE: Mini-Mental State Examination; MoCA: Montreal Cognitive Assessment; BMI: Body Mass Index; TAC: Total Antioxidant Capacity; GSH: Glutathione; IL: Interleukin; TYM: Tell Your Memory; CFT: Categorical Verbal Fluency Test; ADL: Activities of Daily Living; IADL: Instrumental Activities of Daily Living; GAD-7: Generalized Anxiety Disorder scale; MRI: Magnetic Resonance Imaging; VSRAD: Voxel-based Specific Regional Analysis System for Alzheimer’s disease; ADAS-Jcog: Japanese version of the Alzheimer’s Disease Assessment Scale; PSQI: Pittsburgh Sleep Quality Index; GSRS: Gastrointestinal Symptom Rating Scale.

**Table 4:** Probiotic strains and Dosages Administered in Clinical Trials.

Study	Strains of Probiotic/Prebiotic/Synbiotic	Dosage
Agahi et al., 2018 [56]	*L. fermentum*, *L. plantarum*, *B. lactis*, *L. acidophilus*, *B. bifidum*, *B. longum*	3 × 10^9^ CFU/day
Akhgarjand et al., 2022 [57]	*L. rhamnosus* HA and *B. longum* R0175	1 × 10^15^ CFU/day
Asaoka et al., 2022 [58]	*B. breve* MCC127	2 × 10^10^ CFU/day
Fei et al., 2023 [59]	*L. plantarum* BioF-228, *L. lactis* BioF-224, *B. lactis* CP-9, *L. rhamnosus* Bv-77, *L. johnsonii* MH-68, *L. paracasei* MP137, *L. salivarius* AP-32, *L. acidophilus* TYCA06, *L. lactis* LY-66, *B. lactis* HNO19, *L. rhamnosus* HNO01, *L. paracasei* GL-156, *B. animalis* BB-115, *L. casei* CS-773, *L. reuteri* TSR332, *L. fermentum* TSF331, *B. infantis* BLI-02, *L. plantarum* CN201	2 × 10^10^ CFU/g

Notes: L. = Lactobacillus; B. = Bifidobacterium.

## Data Availability

The original contributions presented in the study are included in the article. Further inquiries can be directed to the corresponding author.
