# Correction: Quansah et al. The Beneficial Effects of Lactobacillus Strains on Gut Microbiome in Alzheimer’s Disease: A Systematic Review. *Healthcare* 2025, *13*, 74

**DOI:** 10.3390/healthcare14080974

**Published:** 2026-04-08

**Authors:** Michael Quansah, Monique Antoinette David, Ralph Martins, Emad El-Omar, Silvana Mirella Aliberti, Mario Capunzo, Slade O. Jensen, Mourad Tayebi

**Affiliations:** 1 Neuroimmunology Laboratory, School of Medicine, Western Sydney University, Campbelltown, NSW 2560, Australia; 2 Department of Medicine and Therapeutics, Medical School, University of Ghana, Accra LG25, Ghana; 3 Macquarie Medical School, Faculty of Medicine, Health and Human Sciences, Macquarie University, Macquarie Park, NSW 2109, Australia; 4 Microbiome Research Centre, School of Clinical Medicine, UNSW Medicine & Health, St George & Sutherland Clinical Campuses, UNSW, Kogarah, NSW 2217, Australia; 5 Hygiene and Preventive Medicine Section, Department of Medicine, Surgery and Dentistry “Scuola Medica Salernitana”, University of Salerno, Baronissi, 84081 Salerno, Italy; 6 School of Medicine, Microbiology and Infectious Diseases, Ingham Institute for Applied Medical Research, Western Sydney University, Liverpool, NSW 1871, Australia

In the original publication [1], there were two references cited that have been rectified (previously cited as refs. [51,52]):

51. Huang, H.J.; Chen, J.L.; Liao, J.F.; Chen, Y.H.; Chieu, M.W.; Ke, Y.Y.; Hsu, C.C.; Tsai, Y.C.; Hsieh-Li, H.M. Lactobacillus plantarum PS128 prevents cognitive dysfunction in Alzheimer’s disease mice by modulating propionic acid levels, glycogen synthase kinase 3 beta activity, and gliosis. *BMC Complement. Med. Ther.* **2021**, *21*, 259.

52. Yun, S.W.; Park, H.S.; Shin, Y.J.; Ma, X.; Han, M.J.; Kim, D.H. Lactobacillus gasseri NK109 and Its Supplement Alleviate Cognitive Impairment in Mice by Modulating NF-kappaB Activation, BDNF Expression, and Gut Microbiota Composition. *Nutrients* **2023**, *15*, 790.

With this correction, the order of the references has not changed. The correction does not include referenced data, and the scientific conclusions have not been changed. This correction was approved by the Academic Editor. The original publication has also been updated.
